# First person – Caroline Beltran

**DOI:** 10.1242/dmm.052325

**Published:** 2025-03-26

## Abstract

First Person is a series of interviews with the first authors of a selection of papers published in Disease Models & Mechanisms, helping researchers promote themselves alongside their papers. Caroline Beltran is first author on ‘ Correlative 3D imaging method for analysing lesion architecture in susceptible mice infected with *Mycobacterium tuberculosis*’, published in DMM. Caroline conducted the research described in this article while a postdoctoral researcher in Professor Gerhard Walzl's lab at Stellenbosch University, Cape Town, South Africa, and is now a Scientific Director at Synexa Life Sciences, Cape Town, South Africa, leveraging 3D imaging to decipher host–pathogen interactions and unravel treatment responses in tuberculosis (TB).


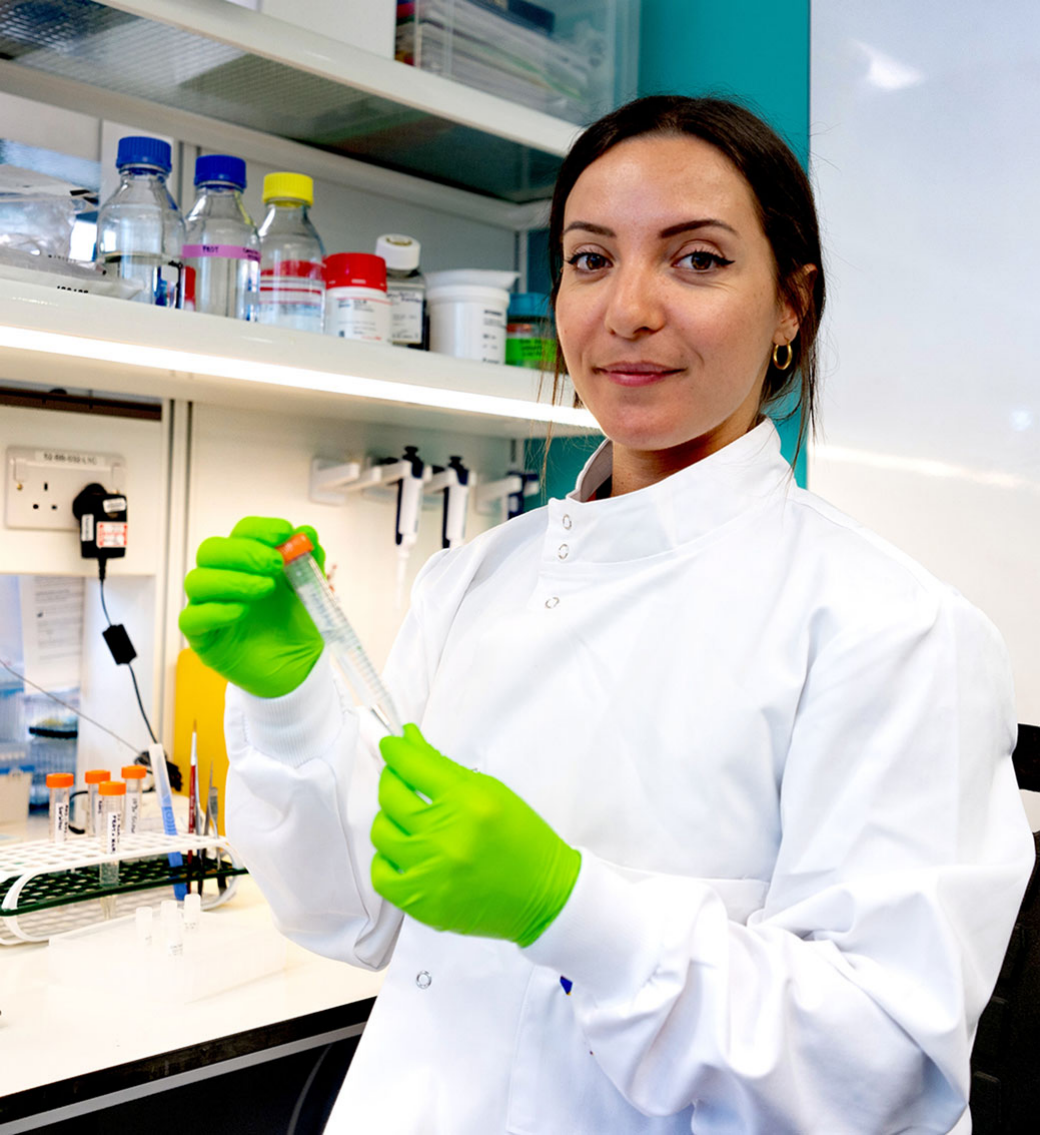


**Caroline Beltran**

**Who or what inspired you to become a scientist?**

I have always been fascinated by the natural world from a young age, when my father bought me a very basic microscope that sparked my love for the micro world. Being able to capture the beauty and complexity that happens in cells led me to pursue a PhD in molecular biology. Living in South Africa, I was acutely aware of the devastating consequences of infectious diseases, which led me to specialize in TB and understanding the complexity of this disease.

**What is the main question or challenge in disease biology you are addressing in this paper? How did you go about investigating your question or challenge?**

TB heterogeneity poses a significant challenge in disease biology, as the variability in lesion architecture, immune cell composition and bacterial distribution complicates our understanding of host–pathogen interactions and treatment responses. In our study, we address this challenge by developing a novel 3D imaging approach that integrates passive CLARITY (PACT)-based tissue clearing with light-sheet fluorescence microscopy (LSFM) and serial block face electron microscopy (SBF-EM). This unique combination enables us to visualize entire lung lobes at multiple time points, revealing the dynamic spatial organization and distinct compartments within TB lesions.

We applied our approach in the C3HeB/FeJ mouse model, which is highly relevant due to its ability to recapitulate the complex lesion types seen in human TB, including necrotic granulomas. By comparing infections with wild-type *Mycobacterium tuberculosis* and an ESX-1 deletion mutant (ΔRD1), we demonstrate how the loss of a key virulence factor alters lesion structure, resulting in reduced immune cell recruitment and less extensive bacterial dissemination. These findings highlight the pivotal role of the ESX-1 secretion system in modulating granuloma architecture and underscore the importance of capturing lesion heterogeneity for understanding disease progression.

By combining LSFM and SBF-EM, our method bridges the gap between large-scale volumetric imaging and high-resolution ultrastructural analysis, providing a comprehensive view of TB pathology. This integrative approach offers valuable insights into how differences in granuloma structure may contribute to treatment outcomes, paving the way for more targeted therapeutic interventions.

**How would you explain the main findings of your paper to non-scientific family and friends?**

Imagine trying to understand a movie by only looking at a single still frame – you'd miss the unfolding story. TB is like a long, complex movie with many different scenes happening at once. Traditional methods only give us a few snapshots, making it hard to see how the entire story develops. Our 3D imaging technique is like being able to watch the full movie, where you can see every scene and how they connect. This approach reveals the variety and complexity within the disease, helping us understand how different parts of the infection change and interact over time.

By capturing the complete architecture of lesions, our approach can help pinpoint specific pathological features that may be targeted by drugs.

**What are the potential implications of these results for disease biology and the possible impact on patients?**

Our method offers a new way to visualize TB lesions in 3D, revealing their full spatial complexity and heterogeneity. This enhanced view not only improves our understanding of disease progression but also makes mouse models – still the most critical tool for testing new therapies – more relevant. By capturing the complete architecture of lesions, our approach can help pinpoint specific pathological features that may be targeted by drugs. Ultimately, this could lead to more effective, tailored treatments and better outcomes for patients.

**Figure:**
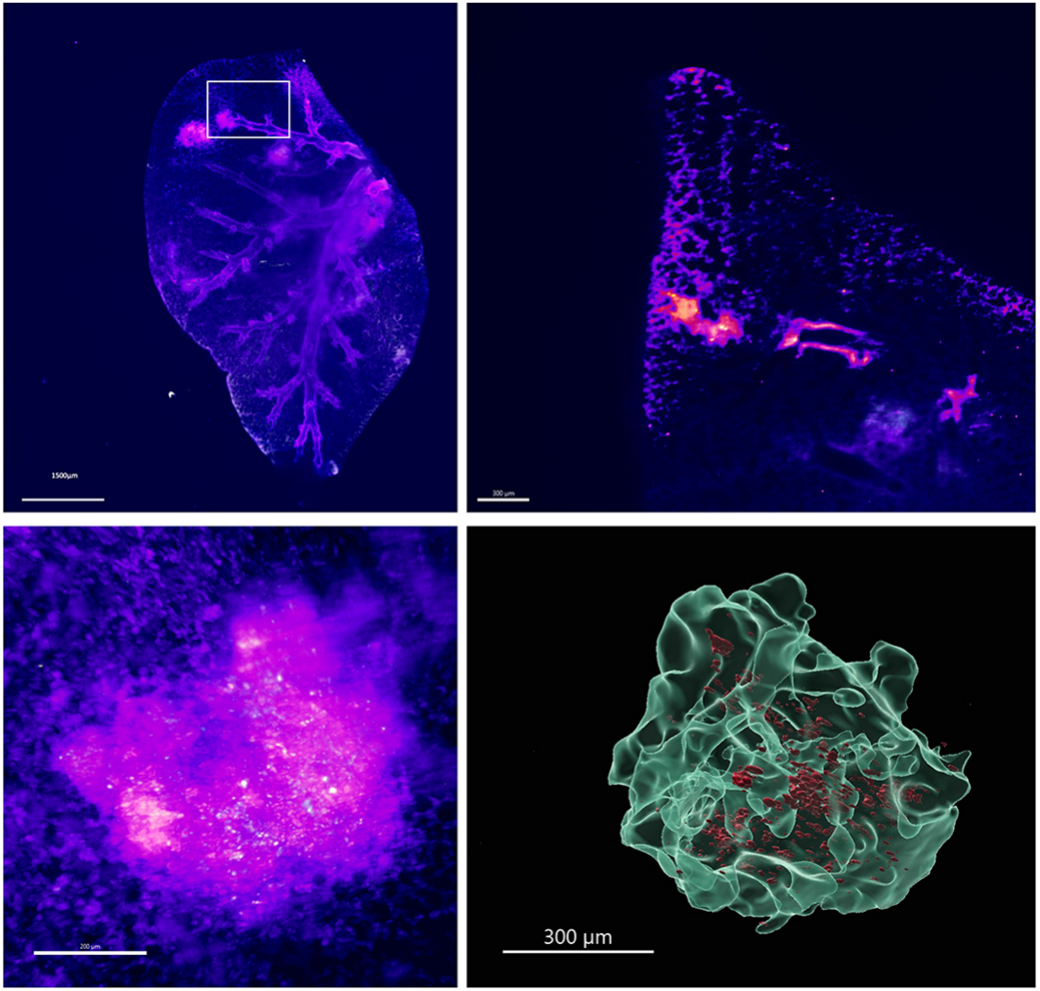
**Still frame from a 3D image of a mouse lung lobe at 28 days post-infection with virulent *M. tuberculosis*, highlighting the onset of a lesion in the upper airways.** Immune cells are shown in pink, *M. tuberculosis* in white. The top-right panel zooms in on a *z*-stack ‘hot zone’ from the top-left panel, illustrating infection dissemination from a branch, while the lower panels provide a 3D view of the lesion, with the final render (bottom left) emphasizing bacterial localization.

**Why did you choose DMM for your paper?**

We chose DMM because it is a highly regarded, open access journal that publishes innovative research on human diseases using diverse model systems. Our work, which focuses on characterizing a TB disease model using cutting-edge 3D imaging techniques to reveal novel mechanistic insights, aligns perfectly with DMM's commitment to advancing the boundaries of traditional approaches. The journal's audience appreciates studies that combine innovative methods with a deep understanding of disease biology, making DMM the natural home for our research.

Being a scientist in Africa has always been a challenge, particularly from a resource point of view.

**Given your current role, what challenges do you face and what changes could improve the professional lives of other scientists in this role?**

Being a scientist in Africa has always been a challenge, particularly from a resource point of view. Balancing complex research projects with administrative and career development tasks is challenging. Greater access to advanced technologies, improved interdisciplinary funding and enhanced mentorship programs significantly benefits early-career scientists. I was lucky for this work to be awarded the Crick African Network award, which allowed me to use the incredible facilities at The Francis Crick Institute to generate the 3D images displayed in our paper. These kinds of initiatives, which allow resource-limited scientists to travel and access highly specialized platforms, are incredibly invaluable. I was also incredibly lucky to have a wonderful mentor at the Crick, Dr Max Gutierrez, who provided excellent support throughout the project.

**What's next for you?**

The 3D work continues – I am currently working as a scientific director, implementing 3D organoids and models for pharmaceutical development.

**Tell us something interesting about yourself that wouldn't be on your CV**

When I am not in the lab, I make ceramics in my garage! Pottery is a great hobby, especially as a scientist where experiments are very rigid. By contrast, creating a piece on the wheel is very free and allows me think creatively, which I bring back to my science.
